# A survey of retracted articles in dentistry

**DOI:** 10.1186/s13104-017-2576-y

**Published:** 2017-07-06

**Authors:** Túlio Eduardo Nogueira, Andréia Souza Gonçalves, Cláudio Rodrigues Leles, Aline Carvalho Batista, Luciane Rezende Costa

**Affiliations:** School of Dentistry, Federal University of Goias, Avenida Universitária, s/n. Setor Universitário, Goiânia, Goiás CEP 74605-220 Brazil

**Keywords:** Ethics, Retracted publication, Plagiarism, Scientific misconduct, Scientific frauds, Duplicate publication, Retracted articles, Bioethics, Dentistry, Retraction of publication

## Abstract

**Background:**

Publication retraction is a mechanism to preserve the scientific literature against publications that contain seriously flawed or erroneous data, redundant publication, plagiarism, unethical research, and other features that compromise the integrity of science. An increase in the occurrence of retractions in recent years has been reported. Nevertheless, there is scarce information on this topic concerning publications in dentistry and related specialties. Thus, this study aimed to investigate retracted papers published in dental journals.

**Methods:**

Data collection included an exploratory search in PubMed and a specific search in SCImago Journal Rank indexed journals, complemented by the cases reported on the Retraction Watch website and in PubMed. All 167 dental journals included in SCImago were searched for identification of retracted articles up to March 2016. The selected retracted articles and their corresponding retraction notices were recorded and assessed for classification according to the reason for retraction and other additional information.

**Results:**

Forty of the 167 journals scrutinised at SCImago (23.9%) had at least one retracted article, and four additional journals were identified from the Retraction Watch website. A total of 72 retracted found were retracted for the reasons: redundant publication (20.8%), plagiarism (18.1%), misconduct (13.8%), overlap (13.6%) and honest error (9.7%). Higher number of retractions were reported in those journals with cites/doc <2.0—n = 49 (74.2%). The types of studies were mainly laboratory studies (34.7%), case reports (22.2%) and review articles (13.9%).

**Conclusions:**

The approach to ethical problems in papers published in dental scientific journals is still incipient; retractions were mostly due to the authors’ malpractice and were more frequently related to journals with less impact.

**Electronic supplementary material:**

The online version of this article (doi:10.1186/s13104-017-2576-y) contains supplementary material, which is available to authorized users.

## Background

According to the United States National Library of Medicine, “articles may be retracted or withdrawn by their authors, academic or institutional sponsor, editor or publisher, because of pervasive error or unsubstantiated or irreproducible data” [1]. For the Committee on Publication Ethics (COPE), a publication retraction allows the literature to be corrected and alerts readers to publications that contain flawed or erroneous data, resulting in findings and conclusions that cannot be reliable [2]. Therefore, it is an action intended to guarantee the integrity of the literature and does not simply focus on penalising “misbehaving” authors [2].

The COPE retraction guidelines recommend that journal editors should consider retracting a publication if clear evidence is found that the findings are unreliable, either as a result of misconduct or honest error, if the findings have previously been published elsewhere without proper cross-referencing, permission or justification, if it constitutes plagiarism, or if it reports unethical research [2].

The increasing movement towards more accountable and transparent science practices has encouraged scientists to improve the way scientific findings are reported and published [3]. However, scientific misconduct still occurs, impacting negatively the credibility of science. The number of retracted articles has increased considerably in recent years [4, 5], although it is unclear whether this is due to the growth in scientific literature or to the greater efficiency in the detection of flawed articles [6, 7]. The effects of retracted papers can be devastating for editors, readers, authors and science, especially considering the incomplete adherence to retraction mechanisms and guidelines [8]. It is not uncommon to find unclear and unhelpful one-line retraction notices, providing no explanation about the reasons for retraction [9], so a study have suggested a standard retraction form to help the editor clarify the reader with any additional information [10]. Moreover, a considerable number of retracted papers continue to be cited even after being retracted [11].

Previous studies investigating characteristics of retracted papers focused on general medicine [12, 13], mental disorders [14], pharmacology [15], radiology [16], and biomedical literature [17], as well as specific topics such as retraction differences across countries [18], scholarly literature in PubMed [10, 19, 20], and noncompliance with human rights in retracted medical papers [11]. However, specific studies about trends and factors associated with retractions in dentistry are still lacking [21].

Systematic identification and assessment of retracted papers in biomedical literature is important to provide information for researchers and readers on this relevant ethical issue, providing guidance and warning against major ethical lapses [22] by thoroughly exploring indexing databases in addition to the “mainstream international publications” [13]. Thus, the aim of this study was to investigate the occurrence of publication retractions in the dental literature and to assess the characteristics of the retracted papers surveyed. The results should alert the dental community to the ethical problems that have occurred in scientific publishing, mobilise publishers and authors to prevent such problems, and help clinicians to avoid the spread of questionable results in the dental literature.

## Methods

The survey was performed using two simultaneous strategies, one exploratory and other more specific for the purpose of this study, detailed as follows.

### Exploratory search

We performed an exploratory PubMed search (updated on March 15, 2016), using the Mesh term “Retracted Publication [Publication type]” without any limit, to have an overview of the number of retracted papers in the biomedical literature indexed in MEDLINE^©^. Then, we added the term “AND dentistry [Mesh]” to this search to identify the retracted articles limited to the dental field. Data obtained were described quantitatively.

### Specific search

This specific strategy included the identification of eligible journals, active contact with journal’s editorial office and electronic article search. We used the SCImago Journal and Country Rank (http://www.scimagojr.com) for identification of dental journals, by selecting the “Journal Rankings” option and using “dentistry” in the “subject area” drop-down list. The SCImago search performed on August 11, 2015, retrieved a list of 167 dental journals, which were considered the primary source for the identification of retracted articles. Additionally, we checked the Retraction Watch website (http://retractionwatch.com) by using the keyword “dentistry” in its search system. Finally, in March 2016, we searched PubMed again for “withdrawn [title] OR retraction [title] OR retracted [title] AND dentistry”, to search for any additional information.

The identification of retracted articles in the 167 selected journals in SCImago involved two approaches. The first consisted of direct contact with the journal’s editorial office, by sending a standardised email requesting the full references of the retracted articles in each journal. We made three contact attempts within a maximum period of 6 weeks. This first approach was very unsuccessful, as we had only 16 clear answers from the journals. Then, the second approach consisted of an electronic search in each journal’s website. We included different keywords in the search, according to the predominant language of the journal. In the case of predominance of the English language, the following terms were used: “retraction of publication”, “retracted publication”, “retraction of articles”, “retraction notice”, “withdrawal”, “retraction” and “retracted”. In Portuguese language journals, we used the terms: “retratação de publicação”, “artigos retratados”, “artigos retirados” and “publicação retratada”. In Spanish language journals, the search was made with “retractación de publicación” and “publicación retractada”. When the search tool was not available, a one-by-one search in the list of contents in each journal’s website was performed. Although the keywords we used were restricted to English, Spanish and Portuguese, no newspaper was excluded because of the original language.

Considering all the search strategies and after obtaining the list of references containing the retracted articles, we looked for each corresponding retraction notice. Based on the content of the notice, the reason of each retraction was classified following specific criteria proposed according to an adaptation of the COPE guidelines [2] (Table 1). If the reason was not mentioned in the retraction notice, it was coded as “no reason reported”. Moreover, additional information regarding the retraction was collected, such as journal’s location of publication, journal’s cites/doc 2 years, study design, study field within dentistry, origin of the retraction’s corresponding author and time between publication of the original article and its retraction.

**Table 1:** Reasons for papers’ retraction adapted from COPE guidelines [2]

Reason	Definition
Redundant publication	Publication of the same data or article in more than one journal without appropriate justification, permission or cross-referencing
Overlap	Some new findings are presented in an article that also contains a substantial amount of previously published information
Misconduct	Evidence of unreliable results caused, for example, by data fabrication
Honest error	Evidence of unreliable results, caused, for example, by a miscalculation or by an experimental error
Plagiarism	Content of other author (data, words or theories) is presented by another author without referencing as it was his own
Authorship issues	Authorship dispute of an article
No reason reported	No clear information of the reasons for the retraction was mentioned

### Data analysis

Data analysis was performed using IBM–SPSS version 20.0 (IBM Corporation, New York, USA, version 20.0) and Graph Prism 5.0 (San Diego, CA, USA) programs.

## Results

### Exploratory search

**Fig. 1:**
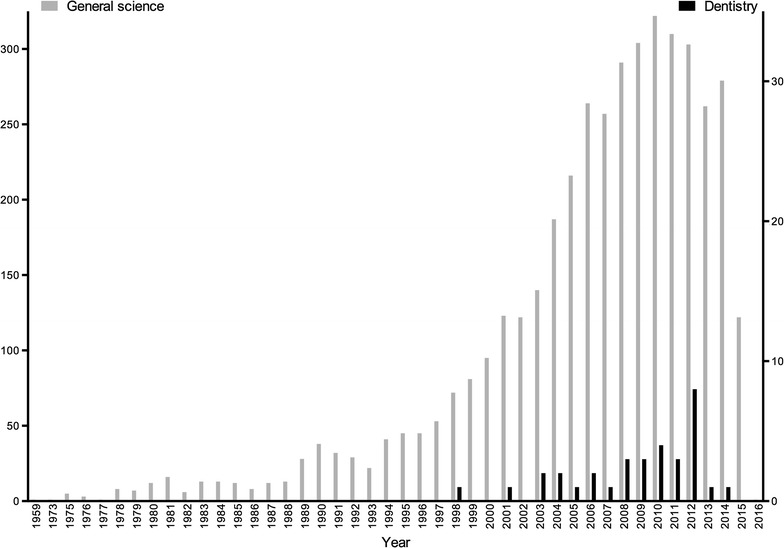
The escalation of retracted papers in PubMed (numbers collected on March 15, 2016)

The exploratory search in PubMed showed that from more than 25 million citations in the biomedical literature, a total of 4215 citations of retracted publications were found. The first citation from a retracted paper was published in 1959, with a peak incidence in 2010 (n = 322) (Fig. 1). Then, by adding the expression “AND dentistry [Mesh]”, we retrieved 33 citations out of a total of 484,468 citations on “dentistry” as a free term (Fig. 1). These data show that retracted papers indexed in PubMed correspond to 0.01% of the biomedical literature as a whole and 0.007% of the dental literature.

### Specific search

We identified a total of 72 retracted articles derived from 44 journals: 40 out of the 167 journals listed in the SCImago portal in the dentistry category (23.9%), complemented by four journals found in the Retraction Watch database. An Additional file 1: Table S1 displays the complete scenario obtained in our investigation.

Considering the 72 retracted articles, redundant publication (n = 15, 20.8%), plagiarism (n = 13, 18.1%), misconduct (n = 10, 13.8%), overlap (n = 9, 13.6%) and honest error (n = 7, 9.7%) accounted for most of the reasons for retraction.

Two-thirds of the corresponding authors of the retracted papers identified in this survey were affiliated with institutions located in India (n = 21, 29.2%), United States (n = 8, 11.1%), China (n = 7, 9.7%), Brazil (n = 5, 6.9%) and Germany (n = 5, 6.9%).

There were 12 journals with more than one retracted article; the higher occurrence of retractions was in the journals Oral Oncology (n = 6; 8.3%) and Dental Materials Journal (n = 5; 6.9%). The highest absolute number of retracted papers in this survey were from journals of the United States (n = 15; 34.1%) and the United Kingdom (n = 12; 27.2%).

**Fig. 2:**
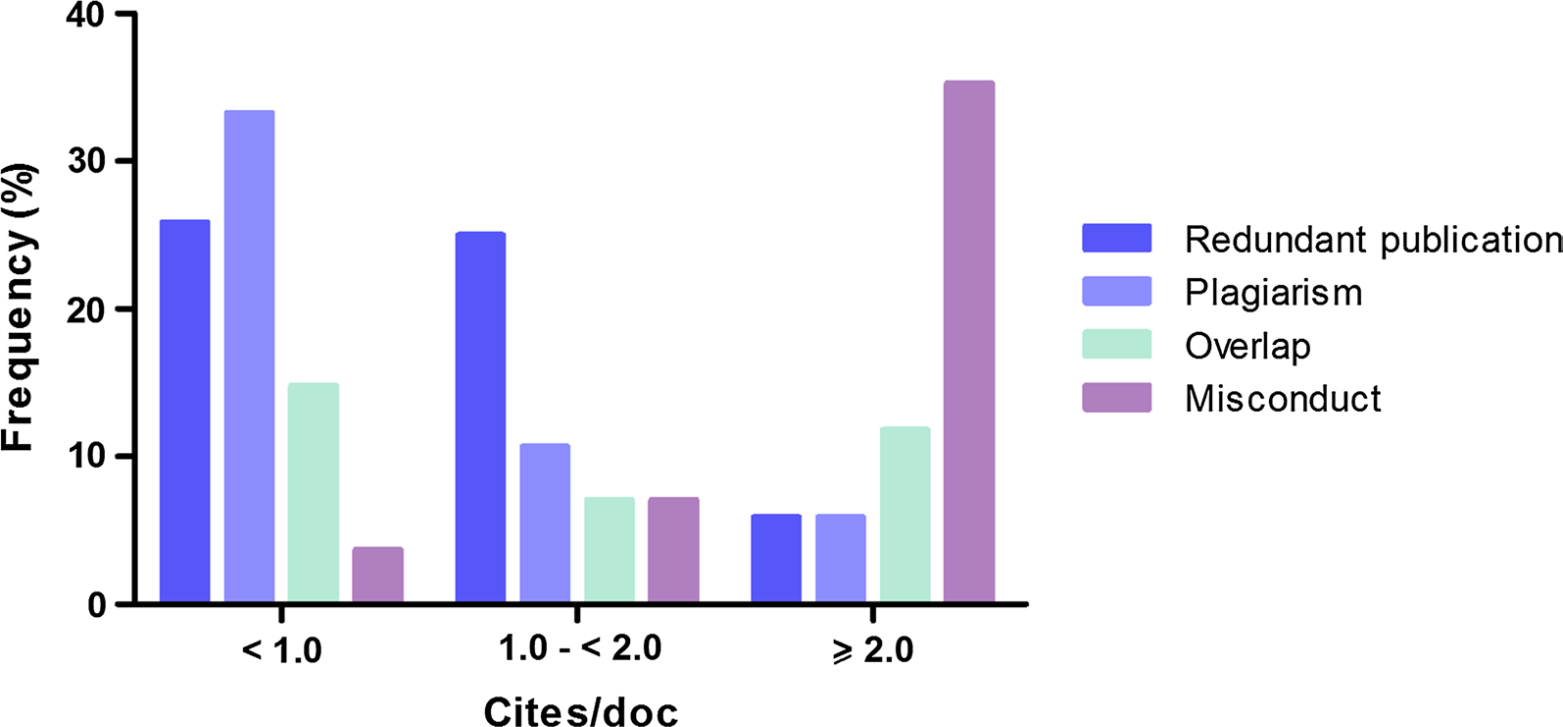
Absolute frequency (n) of the reasons for retraction grouped according to the cites/doc of the journals

After grouping the journals according to the cites/doc index, retractions showed the following distribution: cites/doc <1.0—n = 21 (31.8%); cites/doc between 1.0 and 2.0—n = 28 (42.4%); and cites/doc higher than 2—n = 17 (25.8%) (Fig. 2). Six retractions (8.3%) occurred in journals that are not indexed in SCImago, so they do not have a cites/doc indicator.

**Fig. 3:**
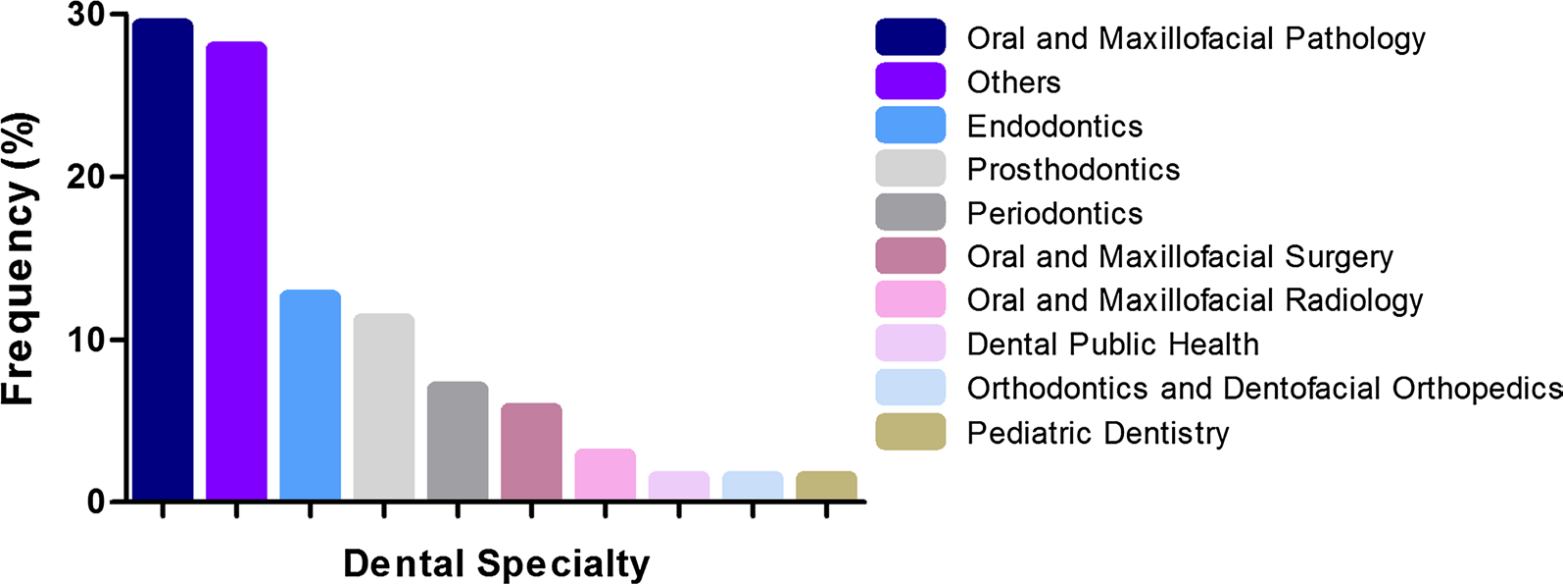
Absolute frequency of retracted articles in different dental specialties

**Fig. 4:**
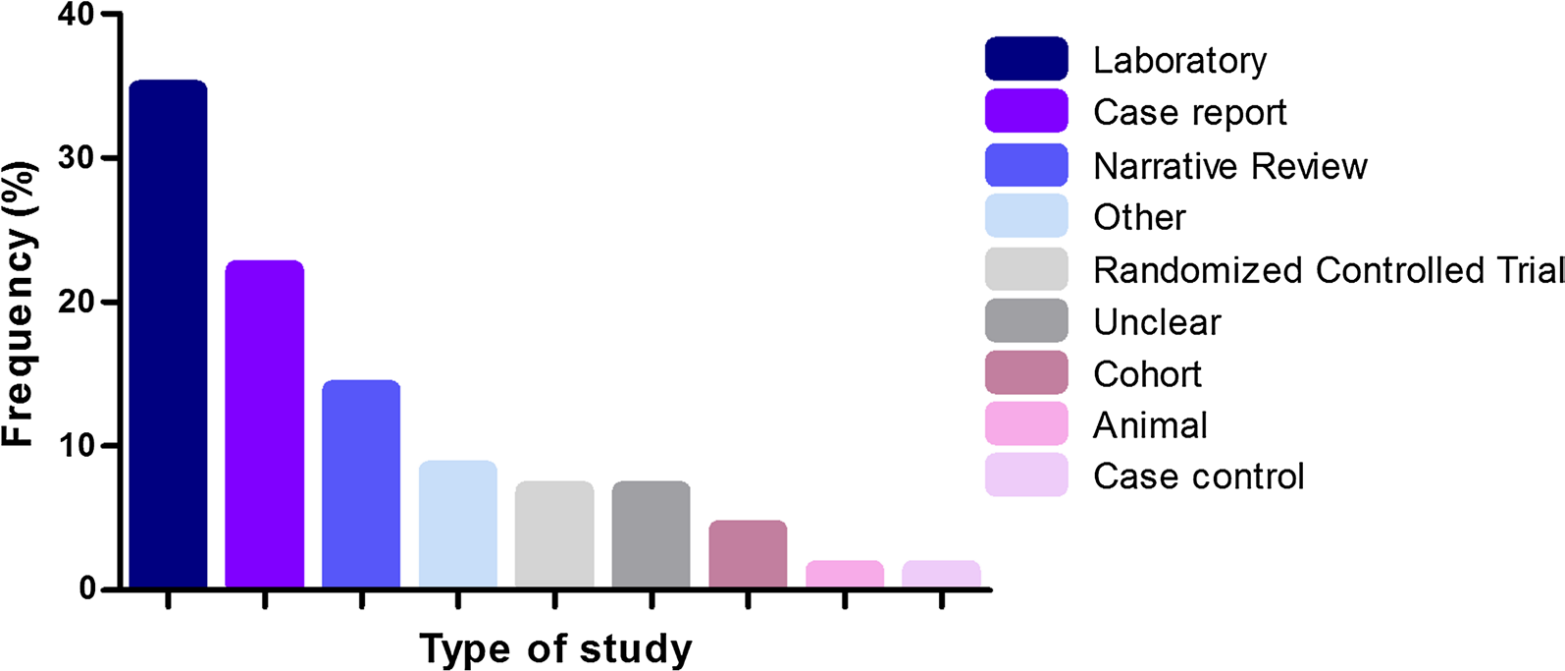
Absolute frequency of retracted articles according to the type of study

The area within dentistry that showed the highest frequency of retractions was Oral and Maxillofacial Pathology (n = 21; 29.2%) (Fig. 3). Regarding the study type, retractions were mainly related to laboratory studies (34.7%), case reports (22.2%) and narrative reviews (13.9%) (Fig. 4).

The period between the publication of the original article and the publication of the retraction notice varied from months to years (median = 10.5 months; minimum = 1 month, maximum = 451 months or 37.6 years).

## Discussion

To our knowledge, this is the first investigation focusing on retracted articles in dentistry and its relevance is evident in the face of the rising number of retractions, which represents a challenge to be faced by the scientific community. Perhaps the most relevant result of this descriptive study is that the proportion of retracted papers in dentistry is low compared to the biomedical literature indexed in PubMed, as revealed by our exploratory findings. We could suggest some reasons for this: there is a time-lag of at least 3 years in PubMed notices of retraction [20]; the retraction of papers in this field of knowledge is a recent occurrence when compared with the biomedical literature as a whole; inappropriate or fraudulent data can be very difficult to detect [23]; dental journals have been slow to endorse well-recognised reporting guidelines [24] that could facilitate the identification of ethical problems in the manuscripts. Since there is lacking information on this topic in the field of dentistry, it is difficult to discuss the findings of our study within the specific context of the dental literature. Hence, comparison across studies and recommendations derived from our findings were presented and discussed within the perspective of the biomedical literature and should be considered in a broader standpoint of ethics and integrity in scientific research.

In the present study, the most observed reasons for retraction, from highest to lowest occurrence, were: redundant publication, plagiarism, misconduct, overlap and honest error. On the other hand, other study that identified 395 retractions in English language publications indexed in MEDLINE^©^ between 1982 and 2002 showed that 61.8% were due to unintentional errors and 27.1% to misconduct [25]. Wager and Williams [4] observed that 28% of retractions were due to honest errors, 17% to redundant publication and 16% to plagiarism. More recently, an analysis of articles retracted in the scholarly literature in 2012 and 2013 demonstrated that the most frequent reasons for retraction were mistakes, plagiarism, and duplicate submission [19]. However, an analysis of 2047 retracted articles, as of May 3, 2012, revealed that 67.4% of them were due to misconduct and 21.3% to error [5]. For these authors [5], misconduct comprised fraud (confirmed or suspected), duplicate (redundant) publication and plagiarism. The lack of uniformity in the definition of ethical issues in relation to the retraction of articles [4] makes more consistent comparisons difficult.

We observed that redundant publication and plagiarism were more reported in journals with cites/doc under 2.0, whilst overlap was more frequent in journals with cites/doc under 1.0. The most observed reason for retraction in journals with cites/doc of 2.0 and above was scientific misconduct. These results are in accordance with the ethical issues found in 2047 retracted notices reported in PubMed [5]. Perhaps journals with smaller cites/doc do not routinely use software to detect plagiarism, redundant publication or overlap. In fact, software such as iThenticate does not effectively detect plagiarism. It detects similarities among texts, so skill is needed to interpret the report [26]. By contrast, journals with higher cite per doc indexes would prevent replication of text early in the process. However, reviewers, editors and the research community would find scientific misconduct more difficult to identify. Interestingly, the higher occurrence of retractions was observed in journals with cites/doc between 1.0 and 2.0.

The issues of redundancy, plagiarism and overlap deserve more discussion, if we consider that publishing a paper is very relevant for authors’ professional advancement. Sometimes, the results of a study may have different implications and outcomes might deserve being reported in several papers [23]. For those cases to be ethically acceptable, authors should disclose the links between that specific report and a wider study, at submission and in citations [23]; if not, that could be seen as “*salami”* publication. Redundant publications, however, are not advisable unless justified by authors and agreed by the editors of both journals. Although, when authors use a methodology that is quite similar to another study already published, clear cross-referencing is advisable to prevent software to identify repeated text as plagiarism. Actually, editors usually suspect plagiarism when a manuscript receives a 35% or higher score for similarity in iThenticate Plagiarism Detection Software [26]. Additionally, there is no agreement among editors regarding accepting papers that were previously presented at a scientific meeting or on the website of an academic institution. Therefore, it is important that authors disclose any prior reporting during the submission process [23].

Another aspect observed in our study was the notable occurrence of unclear retraction notices, which was also reported in a study of all retractions published in 2008 in PubMed under the publication type “retraction of publication” [10]. Besides being unclear, some retractions failed to distinguish between error and misconduct. In addition, retractions can be difficult to find and are not always available on the websites and in the databases of journals [9]. Moreover, attention was drawn to the need to use well-defined criteria, such as the COPE retraction guidelines, which would be important in avoiding improper and unwarranted retractions [2]. As the retraction topic is a relatively recent discussion for the biomedical literature [5], the idea that the retraction process should be extensively clarified among publishers, editors, authors and institutions [2, 8] should also be highlighted in the field of dentistry.

Our investigation also found that most of the retractions resulted from laboratory studies, followed by case reports and review articles. Similar findings were observed in other studies [4, 19], and one group of authors claimed that there is a greater propensity for error in the handling of results in experimental studies [19]. Regardless of the nature of the study, ethics and integrity must be maintained by the researchers, because, as already stated, patient care is at risk [20].

Data extracted in our study showed that retractions do not have country borders regarding the origins of authors and of journals. In the same vein, other studies have found that errors in reported research are a global issue [5, 13]. However, a survey of the general biomedical literature showed that the origin of the authors was related to the reason for retraction: the rates of retractions due to fraud was higher in the United States, German, Japan and China, whilst plagiarism was more common reason in India and China [5].

The time interval between the publication of the original article and the retraction notice varied considerably, ranging from 1 month to 37.6 years. Another study observed a mean time of 2.8 years in 2012, which reduced to 2.2 years in 2013 [19]. In addition, it was reported a gradual upward trend in the time for retraction over time [5]. Interestingly, this is not influenced by the journals’ impact factor [5, 6].

This study has strengths when compared to previous investigations in the biomedical literature. We based our specific search on three databases: SCImago, Retraction Watch and PubMed. SCImago is developed from Scopus, which is a database that indexes a larger number of journals than PubMed, Web of Science and Google Scholar [27]. We also attempted to look for other terms for identification of retraction notices such as “withdrawn” and “retracted” in the title of the publication, and, when the retraction notice was incomplete, we tried to identify the details of the retraction on the Retraction Watch website and other online reports.

One limitation of this study is that we did not follow up the articles retracted. How readers should deal with retracted papers is a debatable issue, especially when considering articles with conclusions of relevant scientific value [5, 10]. Some authors advocate that “improperly obtained” data, even if considered as “valuable” data should not be published, preventing future unethical research [28]. In this context, the decision to continue citing retracted papers is also questionable, since some reasons that lead to the retraction do not necessarily involve the validity of the methodology and then the results might somehow be useful to the scientific community.

We acknowledge that ‘to err is human’ and it is crucial to have the debate about research ethics in dental science reporting disseminated extensively to the different people involved. The ultimate purpose of this paper is to make editors, authors and overall readership aware of the ethical issues related to scientific investigations in dentistry, based on the understanding of the ideal retraction notice as a historical document [29]. Also, it is important to avoid stigmatising authors who made genuine mistakes along with those who have committed misconduct; that justifies the need for clear and standardised retraction notices [4]. Do the present findings represent the tip of the iceberg of ethically questionable publications in dentistry, or have the vast majority of articles in this area been ruled by ethics? The methodology used in this study does not allow for a response. However, our findings, although seemingly mild, and the lack of standardisation in retractions reporting, strongly suggest that researchers in the dentistry field should pay more attention to scientific integrity in their academic routines.

## Conclusion

We conclude that the approach to ethical problems in scientific journals in dentistry is still incipient. More than 50% of cases of retraction in dentistry are represented by the reasons “redundant publication,” “plagiarism” and “misconduct”, by the study types “laboratory research” and “case reports”, and by journals with cites/doc of 2.0 or less.

## Additional file

**Additional file 1: Table S1.** Characteristics of retracted papers.

## Abbreviation

COPE: Committee on Publication Ethics
