# Supplementary material for: A survey of retracted articles in dentistry
Source: BMC Res Notes. 2017 Jul 6;10:253. doi: 10.1186/s13104-017-2576-y (PMC5500970; doi:10.1186/s13104-017-2576-y)
Supplement: Supplementary file 1 — Additional file 1: Table S1. Characteristics of retracted papers. [file 13104_2017_2576_MOESM1_ESM.docx]

Table S1. Characteristics of retracted papers.

| **Journal (Reference #)** | **Journal Country of Publication** | **Cites/doc^1^** | **Reason for retraction^2^** | **Country of the corresponding author** | **Type of study^3^** | **Dental Specialty^4^** | Time interval between original article and the retraction (months) |
| --- | --- | --- | --- | --- | --- | --- | --- |
| 1. Australian Endodontic Journal | United States | 0,72 | Overlap | Brazil | Unclear | Endodontics | 13 |
| 1. BMJ Case Reports | United Kingdom | 0,10 | Overlap | India | Case report | Oral and Maxillofacial Pathology | 5 |
| 1. BMJ Case Reports | United Kingdom | 0,10 | Overlap | India | Case report | Oral and Maxillofacial Pathology | 5 |
| 1. BMJ Case Reports | United Kingdom | 0,10 | Overlap | India | Case report | Oral and Maxillofacial Pathology | 8 |
| 1. Case Reports in Dentistry | Egypt | Not indexed in SCImago | Authorship issues/Misconduct | China | Case report | Oral and Maxillofacial Pathology | 7 |
| 1. Clinical Implant Dentistry and Related Research | United Kingdom | 2,61 | Misconduct | Italy | Randomized Controlled Trial | Prosthodontics | 9 |
| 1. Clinical Oral Implants Research | Denmark | 2,89 | Authorship issues/Honest errors | Germany | Randomized Controlled Trial | Prosthodontics | 5 |
| 1. Contemporary Clinical Dentistry | India | Not indexed in SCImago | Plagiarism | India | Case report | Oral and Maxillofacial Pathology | 15 |
| 1. Contemporary Clinical Dentistry | India | Not indexed in SCImago | Redundant publication | India | Case report | Others | 18 |
| 1. Dental Clinics of North America | United Kingdom | 1,36 | No reason reported | Canada | Narrative Review | Others | 17 |
| 1. Dental Materials Journal | Japan | 1,06 | Other | Greece | Laboratory | Others | 26 |
| 1. Dental Materials Journal | Japan | 1,06 | Other | Taiwan | Laboratory | Others | 77 |
| 1. Dental Materials Journal | Japan | 1,06 | Other | Iran | Laboratory | Others | 23 |
| 1. Dental Materials Journal | Japan | 1,06 | Other | Spain | Laboratory | Others | 16 |
| 1. Dental Materials Journal | Japan | 1,06 | Other | Mahidol | Laboratory | Others | 16 |
| 1. Dentomaxillofacial Radiology | United Kingdom | 1,5 | Overlap | Turkey | Other | Oral and Maxillofacial Radiology | 37 |
| 1. Gerodontology | Denmark | 1,03 | Overlap | India | Other | Dental Public Health | 8 |
| 1. Indian Journal of Dental Research | India | 0,35 | Plagiarism | India | Narrative Review | Periodontics | 18 |
| 1. Indian Journal of Dental Research | India | 0,35 | Plagiarism | India | Other | Oral and Maxillofacial Pathology | 20 |
| 1. Indian Journal of Dental Research | India | 0,35 | Redundant publication | India | Laboratory | Oral and Maxillofacial Radiology | 18 |
| 1. International Endodontic Journal | United Kingdom | 3,34 | Honest error | Brazil | Laboratory | Endodontics | 6 |
| 1. International Endodontic Journal | United Kingdom | 3,34 | Other | Turkey | Randomized controlled trial | Endodontics | 6 |
| 1. International Journal of Dentistry | United States | 1.18 | Plagiarism | Egypt and Saudi Arabia | Cohort | Prosthodontics | 19 |
| 1. International Journal of Stem Cells | South Korea | 0.71 | Author request | Egypt | Laboratory | Oral and Maxillofacial Pathology | 24 |
| 1. International Journal of Prosthodontics | United States | 1.28 | No reason reported | Egypt | Laboratory | Others | 2 |
| 1. International journal of stomatology & occlusion medicine | Austria | Not indexed in SCImago | Plagiarism | India | Narrative Review | Others | 6 |
| 1. Journal of Biomedical Materials Research Part B: Applied Biomaterials | United States | 2.64 | Overlap | Turkey | Laboratory | Others | 30 |
| 1. Journal of Cellular Biochemistry | United States | 3.17 | Overlap | China | Laboratory | Endodontics | 10 |
| 1. Journal of the American Dental Association | United States | 1.50 | Conflict of interest not disclosed | United States | Laboratory | Others | 4 |
| 1. Journal of Contemporary Dental Practice | United States | 0.30 | Plagiarism | Bahrein | Laboratory | Others | 7 |
| 1. Journal of Dental Education | United States | 0.82 | No reason reported | China | Other | Others | 5 |
| 1. Journal of Dental Research | United States | 3.99 | Redundant publication | Iran | Randomized Controlled Trial | Endodontics | 11 |
| 1. Journal of Endodontics | United States | 3.78 | Misconduct | China | Laboratory | Endodontics | 4 |
| 1. Journal of Endodontics | United States | 3.78 | Plagiarism | India | Laboratory | Endodontics | 71 |
| 1. Journal of Esthetic and Restorative Dentistry | United Kingdom | 1.23 | Redundant publication | Germany | Narrative Review | Others | 1 |
| 1. Journal of Indian Prosthodontic Society | India | 0.18 | Redundant publication | India | Narrative Review | Prosthodontics | 35 |
| 1. Journal of Indian Society of Periodontology | India | 0.58 | Plagiarism | India | Other | Periodontics | 5 |
| 1. Journal of Indian Society of Periodontology | India | 0.58 | Redundant publication | India | Case report | Oral and Maxillofacial Surgery | 5 |
| 1. Journal of Indian Society of Periodontology | India | 0.58 | Authorship issues | India | Case report | Oral and Maxillofacial Surgery | 4 |
| 1. Journal of Indian Society of Periodontology | India | 0.58 | Plagiarism | India | Narrative Review | Periodontics | 4 |
| 1. Journal of International Oral Health | India | Not indexed in SCImago | Overlap and Conflict of interest | Saudi Arabia | Cohort | Prosthodontics | 9 |
| 1. Journal of International Oral Health | India | Not indexed in SCImago | Plagiarism | India | Randomized Controlled Trial | Endodontics | 5 |
| 1. Journal of Oral and Maxillofacial Pathology | India | 0.65 | Misconduct | India | Case report | Oral and Maxillofacial Pathology | 16 |
| 1. Journal of Oral and Maxillofacial Surgery | United Kingdom | 1.43 | No reason reported | China | Unclear | Oral and Maxillofacial Surgery | 8 |
| 1. Journal of Oral and Maxillofacial Surgery | United Kingdom | 1.43 | Redundant publication | Brazil | Laboratory | Orthodontics and Dentofacial Orthopedics | 6 |
| 1. Journal of Oral and Maxillofacial Surgery | United Kingdom | 1.43 | Honest error | United States | Case report | Oral and Maxillofacial Pathology | 15 |
| 1. Journal of Oral and Maxillofacial Surgery | United Kingdom | 1.43 | Honest error | United States | Narrative Review | Oral and Maxillofacial Pathology | 32 |
| 1. Journal of Oral Implantology | United States | 1.10 | Redundant publication | United States | Cohort | Prosthodontics | 11 |
| 1. Oral and Maxillofacial Surgery | Germany | 1.16 | Redundant publication | Germany | Case control | Oral and Maxillofacial Surgery | 110 |
| 1. Journal of Orofacial Orthopedics | Germany | 0.78 | Unclear | Germany | Case report | Oral and Maxillofacial Pathology | 451 |
| 1. Journal of Orthodontics | United Kingdom | 0.45 | Redundant publication | Brazil | Laboratory | Prosthodontics | 5 |
| 1. Journal of Periodontology | United States | 2.39 | Honest error | United States | Unclear | Periodontics | 3 |
| 1. Journal of Prosthetic Dentistry | United States | 1.68 | Printer’s fault | United States | Case report | Prosthodontics |  |
| 1. Journal of Prosthetic Dentistry | United States | 1.68 | Plagiarism | United States | Narrative Review | Others | 16 |
| 1. Journal of Prosthetic Dentistry | United States | 1.68 | Unclear | Egypt | Unclear | Others | 1 |
| 1. Journal of Prosthetic Dentistry | United States | 1.68 | Redundant publication | Thailand | Laboratory | Others | 1 |
| 1. Journal of Prosthetic Dentistry | United States | 1.68 | Unclear | Egypt | Unclear | Others | Not available |
| 1. Journal of Prosthodontic Research | Netherlands | 1.72 | Redundant publication | India | Case report | Others |  |
| 1. Journal of Prosthodontics | United Kingdom | 1.13 | Plagiarism | Saudi Arabia | Laboratory | Others | 81 |
| 1. Journal of Tissue Engineering and Regenerative Medicine | United Kingdom | 4.08 | Authorship issues | China | Laboratory | Endodontics | 3 |
| 1. Journal of the California Dental Association | United States | 0.28 | Plagiarism | United States | Narrative Review | Periodontics | 3 |
| 1. Journal of the Indian Society of Pedodontics and Preventive Dentistry | India | 0.39 | Redundant publication | India | Case report | Pediatric Dentistry | 15 |
| 1. Medicina Oral, Patologia Oral y Cirugia Bucal | Spain | 1.46 | Misconduct | Mexico | Animal | Oral and Maxillofacial Pathology | 10 |
| 1. Oral Oncology | United Kingdom | 3.47 | Misconduct | Norway | Other | Oral and Maxillofacial Pathology | 52 |
| 1. Oral Oncology | United Kingdom | 3.47 | Misconduct | Norway | Laboratory | Oral and Maxillofacial Pathology | 45 |
| 1. Oral Oncology | United Kingdom | 3.47 | Misconduct | Norway | Laboratory | Oral and Maxillofacial Pathology | 66 |
| 1. Oral Oncology | United Kingdom | 3.47 | Honest error | Germany | Laboratory | Oral and Maxillofacial Pathology | 6 |
| 1. Oral Oncology | United Kingdom | 3.47 | Honest error | China | Laboratory | Oral and Maxillofacial Pathology | 9 |
| 1. Oral Oncology | United Kingdom | 3.47 | Misconduct | Norway | Narrative Review | Oral and Maxillofacial Pathology | 42 |
| 1. Oral Surgery, Oral Medicine, Oral Pathology and Oral Radiology | United States | 1.36 | Misconduct | Greece | Laboratory | Oral and Maxillofacial Pathology | 69 |
| 1. Oral Surgery, Oral Medicine, Oral Pathology and Oral Radiology | United States | 1.36 | Redundant publication | India | Case report | Oral and Maxillofacial Pathology | 20 |
| 1. Revista Odonto Ciencia | Brazil | 0.1 | Redundant publication | Brazil | Case report | Oral and Maxillofacial Pathology | 13 |

**REFERENCES** [* means that the retraction notice is not available in PubMed.]:

1. de Almeida-Gomes F, Carvalho-Sousa B, Furtado-Leite MC, dos Santos RA, Maniglia-Ferreira C. Retraction: effectiveness of single- versus multiple-visit endodontic treatment of two mandibular central incisors from the same patient. Aust Endod J. 2008 Aug;34(2):76. doi: 10.1111/j.17474477.2007.00070.x.

2. [No authors listed]. Retraction. Maxillary adenomatoid odontogenic tumour. BMJ Case Rep. 2013 Aug 23;2013. pii: bcr2013010004rp. doi: 10.1136/bcr-2013-010004rp.

3. [No authors listed]. Retraction. A rare occurrence of peripheral ossifying fibroma in the first decade of life and its management. BMJ Case Rep. 2013 Aug 23;2013. pii: bcr2013009084rp. doi: 10.1136/bcr-2013-009084rp.

4. [No authors listed]. Retraction. Odontomas--silent tormentors of teeth eruption, shedding and occlusion. BMJ Case Rep. 2013 Aug 29;2013. pii: bcr2012007666rp. doi: 10.1136/bcr-2012-007666rp.

5. [No authors listed]. Retracted: Rhinosporidiosis of the parotid duct. Case Rep Dent. 2014;2014:382142. doi: 10.1155/2014/382142. Epub 2014 Aug 25.

6. [No authors listed]. Retraction. Rehabilitation of the atrophic posterior maxilla using short implants or sinus augmentation with simultaneous standard-length implant placement: a 3-year randomized clinical trial. Clin Implant Dent Relat Res. 2012 Dec;14(6):924.

7. [No authors listed] Retracted: Telescopic crown-retained removable partial dentures on teeth and implants: an 8- to 9-year prospective randomized clinical trial. Clin Oral Implants Res. 2012 Jul;23(7):895. doi: 10.1111/j.1600-0501.2012.02433.x. Epub 2012 Feb 13.

8. [No authors listed]. Unusually large submandibular epidermoid cyst: A case report, differential diagnosis and therapy: Retraction. Contemp Clin Dent. 2015 Jul-Sep;6(3):437. doi: 10.4103/0976-237X.161914.

9. [No authors listed] Retraction notice. Contemp Clin Dent. 2013 Jul;4(3):396.

10. The retraction notice was not found.

11. [No authors listed]. Retraction: Fluoride release and recharge abilities of contemporary fluoride-containing restorative materials and dental adhesives. Dent Mater J. 2015;34(3):410. doi: 10.4012/dmj.2012-144-e.

12. [No authors listed]. Retraction. Effects of welding pulse energy and fluoride ion on the cracking susceptibility and fatigue behavior of Nd:YAG laser-welded cast titanium joints. Dent Mater J. 2013;32(1):195.

13. [No authors listed]. Retraction: A three-dimensional finite element analysis of the effects of restorative materials and post geometry on stress distribution in mandibular molar tooth restored with post-core crown. Dent Mater J. 2014;33(1):147. doi: 10.4012/dmj.2011-138-e.

14. [No authors listed]. Retraction: In vitro osteoinduction of human mesenchymal stem cells in biomimetic surface modified titanium alloy implants. Dent Mater J. 2014;33(1):148. doi: 10.4012/dmj.2012-015-e.

15. [No authors listed]. Retraction: Morinda citrifolia leaves enhance osteogenic differentiation and mineralization of human periodontal ligament cells. Dent Mater J. 2014;33(1):149. doi: 10.4012/dmj.2012-053-e.

16. [No authors listed]. Notice of redundant publication. Dentomaxillofac Radiol. 2013;42(8):20139012. doi: 10.1259/dmfr.20139012.

17. MacEntee M. Retraction. Translation and validation of the Hindi version of the Geriatric Oral Health Assessment Index. Gerodontology. 2012 Sep;29(3):243.

18. [No authors listed]. Retraction notices. Probiotics and prebiotics in periodontal therapy. Indian J Dent Res. 2012 Sep-Oct;23(5):682.

19. [No authors listed]. Mineralized components and their interpretation in the histogenesis of peripheral ossifying fibroma. Indian J Dent Res. 2012 Sep-Oct;23(5):682.

20. [No authors listed]. A color contrast aided density imaging technique to differentiate between dental hard tissues and its relevance. Indian J Dent Res. 2012 Sep-Oct;23(5):682.

21. de Meireles DA, de Brito TC, Marques AA, Garrido AD, Garcia LF, Sponchiado EC Jr. Retracted: Micro-computed tomography evaluation of apical transportation and centring ability of Reciproc and WaveOne systems in severely curved root canals. Int Endod J. 2015 Aug;48(8):814. doi: 10.1111/iej.12436.

22. Bilgili D, Yilmaz S, Dumani A, Yoldas O. Retracted: Postoperative pain after irrigation with Vibringe versus a conventional needle: a randomized controlled trial. Int Endod J. 2016 Aug;49(8):813. doi: 10.1111/iej.12615. Epub 2016 Feb 29.

23. [No authors listed]. Retracted: A Prospective Study of Early Loaded Single Implant-Retained Mandibular Overdentures: Preliminary One-Year Results. Int J Dent. 2013;2013:310726. doi: 10.1155/2013/310726. Epub 2013 Sep 9.

24. Abdel Aziz Aly L, El-Menoufy H, Ragae A, Ahmed Rashed L, Sabry D. Retraction notice: Adipose stem cells as alternatives for bone marrow mesenchymal stem cells in oral ulcer healing. Int J Stem Cells. 2014 Nov;7(2):167. doi: 10.15283/ijsc.2014.7.2.167.

25. [No authors listed]. Article withdrawn. "Influence of resin cements and aging on the fracture resistance of IPS e.max press posterior crowns". Int J Prosthodont. 2012 Mar-Apr;25(2):109.

*26. [No authors listed]. Retraction Note: Aeronautic dentistry: an upcoming branch. J. Stomat. Occ. Med. 2015 Jun;8(2):51.

27. [No authors listed]. Retraction. Push-out bond strengths of two fiber post types bonded with diferent dentin bonding agents. J J Biomed Mater Res B Appl Biomater. 2012 Jul;100(5):1458.

28. [No authors listed]. Retraction: Pro-inflammatory cytokines induce odontogenic differentiation of dental pulp-derived stem cells. X Yang, S Zhang, X Pang, and M Fan. J Cell Biochem 2012 Aug;113(8):2796. doi: 10.1002/jcb.24283.

*29. [No authors listed]. Notice of retraction. J Am Dent Assoc. 2012 Sep;143(9):968-9.

30. [No authors listed]. Retraction notice to: influence of post fit and post length on fracture resistance: an in vitro study. J Contemp Dent Pract 2013;14(3):496-500. J Contemp Dent Pract. 2013 Sep 1;14(5):1008.

*31. Wang G, Tai B, Huang C, Bian Z, Shang Z, Wang Q, Song G. Erratum. Establishing a Multidisciplinary PBL Curriculum in the School of Stomatology at Wuhan University. J Dent Educ. 2008 Oct;72(10):e1214.

32. Giannobile WV. Retraction. A clinical trial of pulpotomy vs. root canal therapy of mature molars. J Dent Res 89:1080-1085. J Dent Res. 2011 Sep;90(9):1145. doi: 10.1177/0022034511417399.

33. [No authors listed]. Retraction notice to mineralized tissue formation by bone morphogenetic protein-7-transfected pulp stem cells: J Endod 38(2012)170-176. J Endod. 2012 Jun;38(6):868.

34. [No authors listed]. Retraction. Comparison of coconut water, propolis, HBSS, and milk on PDL cell survival. J Endod. 2014 Feb;40(2):290. doi: 10.1016/j.joen.2008.01.018.

35. [No authors listed]. Retraction statement: Modern Concepts for Caries Tissue Removal. J Esthet Restor Dent. 2016 Mar-Apr;28(2):136. doi: 10.1111/jerd.12213. Epub 2016 Mar 25.

36. Sujesh M, Rangarajan V, Ravi Kumar C, Sunil Kumar G. Retraction Note to: Stem Cell Mediated Tooth Regeneration: New Vistas in Dentistry. J Indian Prosthodont Soc 2014 Dec;14(Suppl 1):351. doi: 10.1007/s13191-013-0301-7.

37. [No authors listed]. Notice of retraction. J Indian Soc Periodontol. 2012 Oct;16(4):vi.

38. [No authors listed]. Notice of retraction .J Indian Soc Periodontol. 2012 Oct;16(4):vi

39. [No authors listed]. Notice of Retraction. J Indian Soc Periodontol 2014;18:425.

40. [No authors listed]. Neutrophil extracellular traps: Their role in periodontal disease: Retraction. J Indian Soc Periodontol. 2015 Mar-Apr;19(2):128. doi: 10.4103/0972-124X.154336.

41. [No authors listed]. Retraction Notice. J Int Oral Health 2014;6(5):136.

42. [No authors listed]. Retraction Notice. J Int Oral Health 2014;6(4):111.

43. [No authors listed]. Retraction notice. J Oral Maxillofac Pathol. 2012 Sep;16(3):353.

44. [No authors listed]. WITHDRAWN: Presurgical nasoalveolar molding with computer-aided reverse-engineering and rapid prototyping technique in infants with unilateral cleft lip and palate. J Oral Maxillofac Surg. 2011 Nov;69(11):2936. doi: 10.1016/j.joms.2010.06.212. Epub 2010 Dec 31.

45. [No authors listed]. Retraction: Selective use of hand and forearm muscles during bone screw insertion: a natural torque meter. J Oral Maxillofac Surg. 2013 May;71(5):981.

46. Pogrel MA. Decompression and marsupialization as definitive treatment for keratocysts--a partial retraction. J Oral Maxillofac Surg. 2007 Feb;65(2):362-3.

47. Pogrel MA. Decompression and marsupialization as definitive treatment for keratocysts--a partial retraction. J Oral Maxillofac Surg. 2007 Feb;65(2):362-3.

48. [No authors listed]. Retraction. Immediate placement and provisionalization of implant-supported, single-tooth restorations: a retrospective study. J Oral Implantol. 2012 Aug;38(4):434. doi: 10.1563/1548-1336-38.4.434.

49. [No authors listed]. Retraction. Belastungsmessungen nach der Miniplattenosteosynthese von Unterkieferwinkelfrakturen. Oral Maxillofac Surg. 2012 Dec;16(4):405. doi: 10.1007/s10006-012-0362-5.

*50. [No authors listed]. RETRACTED ARTICLE: Hidrotische ektodermale Dysplasie. Fortschritte der Kieferorthopädie. J Orofac Orthoped. 1978 Jul 39(4):292-299.

51. [No authors listed]. Selective use of hand and forearm muscles during mini-implant insertion: a natural torquimeter. J Orthod. 2013 Mar;40(1):89. doi: 10.1179/1465312513Z.00000000059.

52. Quinonez R, Stearns SC. RETRACTED: Issues and early evidence for the economic evaluation of the effects of periodontal therapy on pregnancy outcomes. (J Periodontol 2008;79:203-206). J Periodontol 2008 May;79(5):771. doi: 10.1902/jop.2008.085002.

*53. Rabanal A, Bral M, Goldstein G. RETRACTED: Management of a patient with severe erosive lichen planus in need of an immediate complete denture: A clinical report. J Prosthet Dent. 2007 May;97(5):252-5.

54. [No authors listed]. Retraction notice of "Temporomandibular disorders in relation to female reproductive hormones: a literature review". J Prosthet Dent. 2005 Sep;94(3):305.

55. Saker S, El-Kholany N, Sakrana A, Maria OM. WITHDRAWN: Effect of different dentin cleaning techniques on bond strength and the micromorphology of dentin/self-adhesive resin cement interface. J Prosthet Dent. 2014 Apr 29. pii: S0022-3913(14)00107-3. doi: 10.1016/j.prosdent.2013.12.015. [Epub ahead of print]

56. Khung R, Suansuwan NS. Withdrawn. Duplicate: Effect of gold sputtering on the adhesion of porcelain to cast and machined titanium. J Prosthet Dent. 2013 Aug;110(2):101-6. doi: 10.1016/S0022-3913(13)60348-0.

57. Saker S, El-Kholany N, El-Gendy A, Fadhil ON, Maria OM. WITHDRAWN: Effect of post space conditioning and luting resin on the retentive strength of fiber-reinforced composite resin posts. J Prosthet Dent. 2014 Apr 22. pii: S0022-3913(14)00110-3. doi: 10.1016/j.prosdent.2013.07.030. [Epub ahead of print]

*58. [No authors listed]. WITHDRAWN: Prosthodontic rehabilitation in Sjogren's syndrome with a simplified palatal reservoir:Two year follow up. J Prosthodont Res. 2011 Oct;55(4):248-51. doi: 10.1016/j.jpor.2011.02.001

59. [No authors listed]. Retraction statement. Effect of chemical disinfectants and repair materials on the transverse strength of repaired heat-polymerized acrylic resin. J Prosthodont. 2013 Jun;22(4):341.

60. [No authors listed]. Retracted: Effects of pro-inflammatory cytokines on mineralization potential of rat dental pulp stem cells. J Tissue Eng Regen Med. 2011 Oct;5(9):759. doi: 10.1002/term.444. Epub 2011 Jul 11.

61. Felsenfeld AL, Carney KK. Retraction: Panaite D, Klokkevold P, and Charles A. The peri-implant papilla: realities on papilla preservation and reformation. J Calif Dent Assoc 36(11):851-67, 2008. J Calif Dent Assoc 2009 Feb;37(2):71.

62. [No authors listed]. Retraction notice. J Indian Soc Pedod Prev Dent. 2012 Jul-Sep;30(3):282.

63. [No authors listed]. Retraction: erbB expression changes in ethanol and 7,12- dimethylbenz (a)anthracene-induced oral carcinogenesis. Med Oral Patol Oral Cir Bucal. 2013 Mar 1;18(2):e325-31. Med Oral Patol Oral Cir Bucal. 2014 Jan 1;19(1):e98.

64. [No authors listed]. Retraction notice to 'When is an oral leukoplakia premalignant?' [Oral Oncol. 38 (2002) 813-814]. Oral Oncol. 2007 Apr;43(4):419.

65. [No authors listed]. Retraction notice to ‘Cyclooxygenase-2 (COX-2) expression in high-risk premalignant oral lesions’ [Oral Oncol. 39 (2003) 497–505]. Oral Oncol. 2007 Apr;43(4):420.

66. [No authors listed]. Retraction notice to ‘Abnormal DNA content predicts the occurrence of carcinomas in non-dysplastic oral white patches’ [Oral Oncol. 37 (2001) 558–565]. Oral Oncol. 2007 Apr;43(4):418.

67. [No authors listed]. Retraction notice to ‘‘Nef from SIVmac239 decreases proliferation and migration of adenoid-cystic carcinoma cells and inhibits angiogenesis’’ [OO 47 (2011) 847–854]. Oral Oncol. 2012 Jan;48(1):95.

68. Peng JP, Chang HC, Hwang CF, Hung WC. Retraction to ‘‘Overexpression of cyclooxygenase-2 in nasopharyngeal carcinoma and association with lymph node metastasis’’ [Oral Oncol. 41 (2005) 903–908]. Oral Oncol. 2006 Jul;42(6):653.

69. [No authors listed]. Retraction notice to ‘Molecular based treatment of oral cancer’ [Oral Oncol. 39 (2003) 749–758]. Oral Oncol. 2007 Apr;43(4):421.

70. Nikitakis NG, Scheper MA, Papanikolaou VS, Sauk JJ. Retracted: The oncogenic effects of constitutive Stat3 signaling in salivary gland cancer cells are mediated by survivin and modulated by the NSAID sulindac. Oral Surg Oral Med Oral Pathol Oral Radiol. 2014 Dec;118(6):746.

71. [No authors listed]. Retraction notice. Oral Surg Oral Med Oral Pathol Oral Radiol Endod. 2008 Sep;106(3):463.

*72. [No authors listed]. Retraction: Burkitt's lymphoma: clinic progression and prognosis. Two different cases reports in young patients [Rev. odonto ciênc. 2010;25(4):417-421]. Rev Odonto Cienc 2012;27(3) 190.
